# The carcinogenic potential of tacrolimus ointment beyond immune suppression: a hypothesis creating case report

**DOI:** 10.1186/1471-2407-6-7

**Published:** 2006-01-11

**Authors:** Jürgen C Becker, Roland Houben, Claudia S Vetter, Eva B Bröcker

**Affiliations:** 1 Department of Dermatology, Julius-Maximilians-University, Würzburg, Germany

## Abstract

**Background:**

Since tacrolimus ointment was approved by the U.S. Food and Drug Administration (FDA) as a promising treatment for atopic dermatitis, it has been approved in more than 30 additional countries, including numerous European Union member nations. Moreover, in the current clinical routine the use of this drug is no longer restricted to the approved indication, but has been extended to a wide variety of inflammatory skin diseases including some with the potential of malignant transformation. So far, the side-effects reported from the topical use of tacrolimus have been relatively minor (e.g. burning, pruritus, erythema). Recently, however, the FDA reviewed the safety of topical tacrolimus, which resulted in a warning that the use of calcineurin inhibitors may be associated with an increased risk of cancer.

**Case presentation:**

Oral lichen planus (OLP) was diagnosed in a 56-year-old women in February 1999. After several ineffective local and systemic therapeutic measures an off-label treatment of this recalcitrant condition using Tacrolimus 0.1% ointment was initiated in May 2002. After a few weeks of treatment most of the lesions ameliorated, with the exception of the plaques on the sides of the tongue. Nevertheless, the patient became free of symptoms which, however, reoccurred once tacrolimus was weaned, as a consequence treatment was maintained. In April 2005, the plaques on the left side of the tongue appeared increasingly compact and a biopsy specimen confirmed the suspected diagnosis of an oral squamous cell carcinoma.

**Conclusion:**

The suspected causal relationship between topical use of tacrolimus and the development of a squamous cell carcinoma prompted us to test the notion that the carcinogenicity of tacrolimus may go beyond mere immune suppression. To this end, tacrolimus has been shown to have an impact on cancer signalling pathways such as the MAPK and the p53 pathway. In the given case, we were able to demonstrate that these pathways had also been altered subsequent to tacrolimus therapy.

## Background

Tacrolimus is the generic name for the macrolide immunosuppressant previously known by its experimental name FK506 [1]. Tacrolimus was first discovered while screening for antibacterial activity of a multitude of compounds. This macrolide is produced by *Streptomyces tsukabaensis*, a bacterium found in the soil near Tsukuba, Japan. The mechanism of action of tacrolimus is closely related to that of cyclosporine. However, while tacrolimus binds tightly to the cellular protein named FKBP (FK506-binding protein) 12, cyclosporine binds cyclophilin. The target of either drug/intracellular receptor complex is a calcium-activated phosphatase called calcineurin, which is required for many functions in a variety of tissues: learning and memory, renal function, and immune responses. The selective sensitivity of immune function to these drugs has two reasons: 1. the low level of expression of calcineurin in lymphocytes relative to cells in other tissues; 2. an absolute requirement for calcineurin in immune activation. During antigen specific T-cell activation intracellular calcium is released and calcineurin is activated to dephosphorylate its target proteins including the transcription factor NFAT (*n*uclear *f*actor of *a*ctivated *T *cells). Upon dephosphorylation, NFAT translocates to the nucleus, where it binds its nuclear counterpart to form an active transcription factor inducing the production of several cytokines mandatory for initiating an immune response. Hence, calcineurin inhibitors interfere with antigen specific T-cell activation. Furthermore, tacrolimus affects the function of mast cells, basophile leucocytes and Langerhans cells. These characteristics explain the great interest to apply tacrolimus topically on inflamed skin, particularly since it was the first new topical immune suppressant since the introduction of steroids. The first successful use of topical tacrolimus in patients with atopic dermatitis was reported by Nakagawa et al. in 1994 and already 6 years later the U.S. Food and Drug Administration (FDA) approved tacrolimus ointment as a promising treatment for atopic dermatitis [2,3]. Additionally, tacrolimus was investigated for a wide variety of inflammatory skin diseases beyond atopic dermatitis; particularly for conditions recalcitrant to other forms of therapy.

Lichen planus is a relatively common disorder, estimated to affect 0.5% to 2.0% of the general population. It is a chronic, inflammatory disease that affects mucosal and cutaneous tissues. Oral lichen planus (OLP) occurs more frequently than the cutaneous form, and while cutaneous lesions in the majority of patients are self-limiting and mainly cause pruritus, oral lesions are chronic, rarely undergo spontaneous remission, and are a potential source of significant morbidity [4]. Several clinical forms of OLP have been described, but in general three types of lesions can be distinguished: reticular, including white lines, plaques and papules; atrophic or erythematous; and erosive, including ulcerations and bullae. The classic histopathologic features of OLP include liquefaction of the basal cell layer accompanied by apoptosis of the keratinocytes and a dense band-like lymphocytic infiltrate at the interface between the epithelium and the connective tissue. In addition, focal areas of hyperkeratinized epithelium, which give rise to the clinically apparent Wickham's striae, and occasional areas of atrophic epithelium where the rete ridges may be shortened and pointed result in a saw tooth appearance. Finally, eosinophilic colloid bodies representing degenerated keratinocytes, in the lower half of the surface epithelium are typical. Whereas reticular lesions are generally asymptomatic and often discovered incidentally during an oral examination, erythematous and erosive lesions frequently result in discomfort, causing patients to seek care. Unfortunately, oral lesions are difficult to palliate. Indeed, meta-analyses provided little evidence for the superiority of any assessed interventions over placebo for palliation of symptomatic OLP [4]. Therefore it is important that several recent reports suggest the long-term efficacy and safety of topical tacrolimus for the treatment of OLP in more than 100 patients [5-9].

## Case presentation

**Figure 1:**
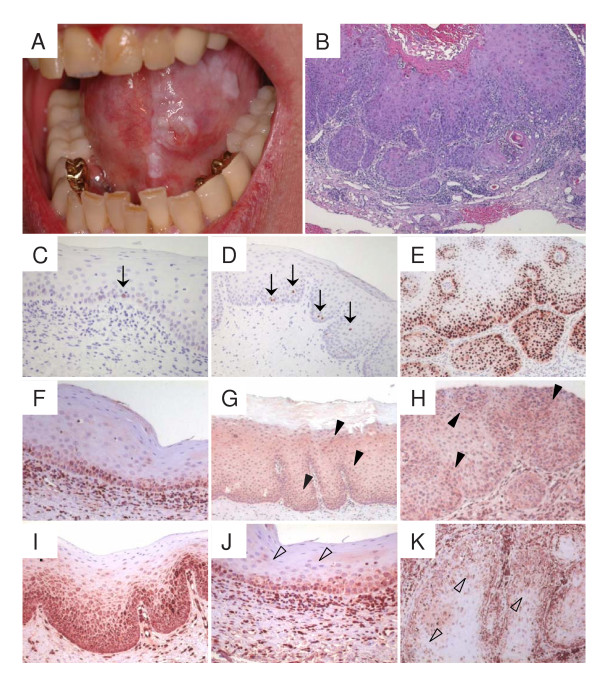
**Macroscopic, microscopic and immune histological appearance of an oral lichen planus and a subsequently arising squamous cell carcinoma**. Macroscopic (A) and microscopic (B) picture of the squamous cells carcinoma. p53 expression (C to E, single positive cells are indicated by arrows), Erk 1/2 phosphorylation (F to H, increased expression is indicated by closed triangles) and Bax expression (I to K, reduced expression is indicated by open triangles) in mucosa before (C, F, I) and after (D, G, J) tacrolimus treatment, as well as in the arising squamous cell carcinoma (E, H, K); magnification: A 5×, C to K 20×. All lesions were obtained by surgical excision, fixed in formalin and embedded in paraffin. Five μm sections of tumor lesions were fixed in acetone and air dried for 30 min. Slides were incubated for 30 min with the indicated specific primary antibodies (anti-p53 [clone D07] and anti-Bax [polyclonal], DAKO, Hamburg, Germany; anti-pErk 1/2 [clone E10], Cell Signalling, BioLabs New England, Frankfurt, Germany) at predetermined dilutions ranging from 1:200 to 1:800.

A 59-year-old women was seen in February 1999, presenting interlacing white keratotic lines with an erythematous border located bilaterally on the buccal mucosa and the palate. Additional lesions were found on both sides of the tongue; the latter were of plaque-like form and resembled leukoplakia. Particularly the plaque-like lesions were associated with a moderate burning sensation. Upon further clinical examination, additional genital lichen planus lesions involving the introitus vaginae were detected. Two biopsy specimens were taken, which established the diagnosis of OLP. Tests for hepatitis B and C were negative; laboratory analyses revealed no pathological findings. Mycology swabs did not show significant growth of *candida albicans*. However, the patient received sympathicolytics for anti-hypertensive therapy. In the following, the sympathicolytics were substituted by Ca-antagonists. After contra-indicating conditions were ruled out, the patient received systemic dapsone therapy in combination with vitamine E. Locally, mometasone furoate monohydrate cream was applied twice daily. These measures largely palliated the symptoms and the clinical aspect was improved, but lesions never cleared completely. After approximately one year without any further improvement, dapsone therapy was discontinued and new biopsy specimens were taken. The histological workup, however, was not specific, but a bullous autoimmune disease could be excluded. In July 2001, systemic acitretin at 0.5 mg/kg body weight was started, but stopped within 6 weeks due to elevated serum lipids. Subsequent biopsy specimens of the genital and oral mucosa were consistent with lichen planus. Due to severe symptoms interfering with normal masticatory function, systemic high dose dexamethasone (100 mg on three consecutive days every 4 weeks) was administered three times, unfortunately without any success. As a consequence, in May 2002 an off-label treatment of the recalcitrant OLP was initiated. Tacrolimus 0.1% ointment (Protopic^® ^0.1%) was applied twice daily. Substantial pain relief was reported after a few weeks of treatment and most of the lesions ameliorated, with the exception of the plaques on the sides of the tongue. After reduction of the frequency of treatment, a recurrence was observed with an increasing number of ulcerated lesions. Consequently, tacrolimus 0.1% was again administered twice daily. In October 2003 an additional biopsy specimen was taken of the ulcerated plaque on the side of the tongue confirming the diagnosis of OLP without evidence of neoplastic transformation. Albeit the OLP did not resolve by treatment with tacrolimus, the patient remained free of symptoms. Hence, the therapy was maintained; however, it was attempted to decrease the frequency of tacrolimus administration. In April 2005, the plaques on the left side of the tongue appeared increasingly compact and a biopsy specimen confirmed the suspected diagnosis of an oral squamous cell carcinoma (Figure 1A and 1B). After exclusion of systemic metastases, a combination of radiation and chemotherapy was initiated to be followed by surgery.

The pre-malignant potential of OLP has been a controversial issue for the past several decades. Indeed, the reported transformation rates vary from 0 to 9% [4,10,11]. Some of the controversy can be attributed to the fact that several studies have focused on the development of oral cancer in cohorts of patients with OLP diagnosed on the basis of different criteria and followed for various periods of time. Despite these differences, the majority of studies have reported a rate of malignant transformation of OLP between 0.5 and 2% over a five year period. Unfortunately, no obvious specific clinical features unequivocally predict the potential for cancer development. In this respect it should be noted, that the notion that ulcerative/erosive OLP lesions are more prone to develop into cancer could not be confirmed. A recent meta-analysis revealed that the different types of OLP developing oral cancer have a rather equal distribution: reticular 33%, plaque 29%, atrophic 13%, and ulcerative/erosive 25% [11]. It has also been proposed, that otherwise benign appearing lesions showing an over-expression of p53 in some of the cells implies the expression of mutated p53 which may be an indicator of potential malignant development. Notably, retrospective analysis of the biopsy specimens revealed such accumulation of p53 over-expressing cells in small clusters (Figure 1C and 1D).

The risk of using tacrolimus topically with respect to carcinogenesis has been of some concern. The reason for this is the fact that systemic long-term treatment with tacrolimus in organ transplant recipients increases the incidence of malignant tumours, particularly squamous cell carcinoma [12]. Recently, there was a first report of the development of a squamous cell carcinoma of the penis after topical use of tacrolimus [13]. In March 2005 the FDA has issued a public health advisory to inform healthcare professionals and patients about a potential cancer risk from the use of tacrolimus which was based on animal studies and case reports in a small number of patients [14]. Causative associations are uncertain, but several patients in whom cancer developed after drug use have been reported. For tacrolimus, 19 cases of cancer were reported, involving 16 adults and 3 children under the age of 16. The cancers were diagnosed 21–790 days after the start of therapy. Nine cases involved lymphomas, and 10 involved skin tumours. The majority of the skin tumours occurred at the site of the drug application. Tumour types included squamous cell carcinoma, cutaneous sarcoma and malignant melanoma. The mechanism is thought to be an inhibition of immune competent cells, which normally survey and prevent malignant and pre-malignant cells from developing into malignant tumours.

**Figure 2:**
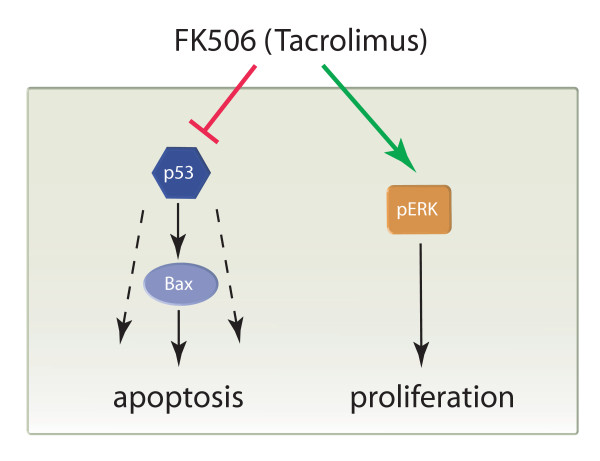
Hypothetical effects of tacrolimus in keratinocytes promoting oncogenic transformation.

It was shown that tacrolimus accelerates carcinogenesis in mouse skin when applied topically after the skin had been pre-treated with a tumour initiator (DMBA) [15]. Because of a reduction in the CD4/CD8 ratio found in the lymph nodes in tacrolimus-treated mice, the authors concluded that the immunosuppressive effect of the drug was responsible for its effect in promoting tumourigenesis. This notion was further substantiated by the finding that the concentration of tacrolimus in the draining lymph node was as high as lymph nodes of animals receiving oral tacrolimus, despite the fact that the serum concentration of tacrolimus in topically treated mice was 50- to 100-fold lower [16]. It should be further noted, that despite of an augmentation of apoptosis in T-cells, tacrolimus was also shown to inhibit apoptosis in non-lymphoid cells [17-20]. Moreover, an influence on proteins of some of the most significant cancer signalling pathways (e.g. Erk and p53) has been demonstrated (Figure 2) [19-22]. Consequently, the carcinogenic potential of tacrolimus may be also due to an direct effect, promoting the transformation of initiated cells.

Treatment with tacrolimus leads to Erk activation in neuronal cells and inhibits the induction of p53 following an apoptotic stimulus in several cell systems [19-22]. In the present case, we actually observed an increased presence of phosphorylated Erk 1/2 within the mucosal epithelium after tacrolimus therapy (Figure 1F and 1G). Moreover, strong phosphoErk signals were obvious in the cells of the tongue carcinoma (Figure 1H). Since the expression levels of p53 are difficult to interpret, as in many tumours p53 accumulates as an inactive mutated protein, we also analysed the expression level of Bax (Figure 1E and 1I to 1K). Bax is a proapoptotic member of the Bcl-2 family and its transcription is directly regulated by p53; hence it may serve as a read out of p53 function. Moreover, it was already reported that tacrolimus prevented an increase in the Bax/Bcl-2 ratio following an apoptotic stimulus in U251 cells, while others found a reduction in mitochondrial Bax with the overall expression level remaining unchanged [19,20]. Strikingly, we observed a reduction of Bax expression in epithelial cells in some areas of tacrolimus treated mucosa and this reduction was also present in the carcinoma cells (Figure 1I to 1K). It should be further noted, that an even more direct link between tacrolimus and Bcl-2 family proteins results of the finding that the tacrolimus-binding protein FKBP 38 blocks apoptosis, binds to Bcl-2 and targets Bcl-2 to the mitochondria [17].

## Conclusion

Taken together, previous reports and our observation suggest that a carcinogenic potential of tacrolimus might also be mediated via direct effects thereby promoting oncogenic transformation of initiated cells. We are fully aware that this is a rather hypothetical scenario and we sincerely hope that the future will prove it wrong. However, it may take human studies of ten years or longer to determine if use of tacrolimus is linked to cancer [23,24]. In the meantime, we hope that this report may raise some concern about the potential carcinogenic effect of tacrolimus and similar compounds which goes beyond indirect mechanisms caused by immune suppression. Moreover, it should be kept in mind that innovative and effective drugs which interfere with intracellular signalling are likely to have more impact on the cell than initially anticipated.

## Abbreviations

FDA – Food and drug administration; OLP – oral lichen planus; NFAT – nuclear factor of activated T cells; FKBP – FK506-binding protein;

## Competing interests

The author(s) declare that they have no competing interests.

## Authors' contributions

JCB, RH and EBB were responsible for the conceptual design of the work, JCB and CSV preformed and analyzed the immune histology, JCB wrote the manuscript, which was corrected and approved by all authors.

## Pre-publication history

The pre-publication history for this paper can be accessed here:
